# Functional characterization of a *csoR-cueA* divergon in *Bradyrhizobium liaoningense* CCNWSX0360, involved in copper, zinc and cadmium cotolerance

**DOI:** 10.1038/srep35155

**Published:** 2016-10-11

**Authors:** Jianqiang Liang, Mingzhe Zhang, Mingmei Lu, Zhefei Li, Xihui Shen, Minxia Chou, Gehong Wei

**Affiliations:** 1 State Key Laboratory of Crop Stress Biology in Arid Areas, College of Life Sciences, Northwest A&F University, Yangling, Shaanxi, China

## Abstract

Random mutagenesis in a symbiotic nitrogen-fixing *Bradyrhizobium liaoningense* CCNWSX0360 (Bln0360) using Tn*5* identified five copper (Cu) resistance-related genes. They were functionally sorted into three groups: transmembrane transport (*cueA* and *tolC*); oxidation (*copA*); and protection of the membrane barrier (*lptE* and *ctpA*). The gene *cueA*, together with the upstream *csoR* (Cu-sensitive operon repressor), constituted a *csoR-cueA* divergon which plays a crucial role in Cu homeostasis. Deletion of *cueA* decreased the Cu tolerance of cells, and complementation of this mutant restored comparable Cu resistance to that of the wild-type. Transcriptional and fusion expression analysis demonstrated that *csoR-cueA* divergon was up-regulated by both the monovalent Cu^+^ and divalent Zn^2+^/Cd^2+^, and negatively regulated by transcriptional repressor CsoR, via a bidirectional promoter. Deletion of *csoR* renders the cell hyper-resistant to Cu, Zn and Cd. Although predicted to encode a Cu transporting P-type ATPase (CueA), *cueA* also conferred resistance to zinc and cadmium; two putative N-MBDs (N-terminal metal binding domains) of CueA were required for the Cu/Zn/Cd tolerance. Moreover, *cueA* is needed for nodulation competitiveness of *B. liaoningense* in Cu rich conditions. Together, the results demonstrated a crucial role for the *csoR-cueA* divergon as a component of the multiple-metal resistance machinery in *B. liaoningense*.

Some transition metals, such as copper (Cu) and zinc (Zn), are essential for many cellular processes, however, they are also toxic in excess by generating free radical species or displacing other metals from their native binding sites in metalloenzymes^1^. Cadmium (Cd), a ubiquitous metal with unknown biological function, can be extremely toxic, even at low levels. Thus, bacteria have evolved various mechanisms to control intracellular metal ion concentrations, ensuring that they do not reach toxic levels.

Mechanisms of Cu resistance and their regulation have been studied extensively in model organisms *Escherichia coli*, *Enterococcus hirae* and *Mycobacterium tuberculosis*. In *E. coli*, detoxification of intracellular Cu is primarily accomplished by Cu transporting P-type ATPase (CopA), multicopper oxidase (CueO) and resistance-nodulation-cell division (RND)-type Cus system (CusCFBA)^2^. However, in Gram-positive *E. hirae*, the Cus system and CueO are absent, and Cu homeostasis mainly depends on the Cop system comprising a transcriptional repressor (CopY), a Cu chaperone (CopZ), and two Cu transporting P-type ATPases (CopA and CopB)^3^. In *M. tuberculosis*, CtpV, a Cu transporting P-type ATPase and MctB, a Cu transport outer membrane protein, together with metallothionein MymT, constitute the defense system against excess Cu^4^.

Although there are a variety of Cu resistance mechanisms, active efflux mediated by Cu transporting P_1B_-type ATPase is the most central^4^. The P_1B_-type ATPases confer heavy metal resistance through pumping out cytoplasmic metal ions including Ag^+^, Cu^+^, Cu^2+^, Zn^2+^, Cd^2+^, Pb^2+^, Co^2+^, Fe^2+^, and Ni^2+^. They are divided into five groups according to their substrate specificity (P_1B1_–P_1B5_), among which P_1B1_- and P_1B2_-type ATPases are responsible for Cu^+^/Ag^+^ and Zn^2+^/Cd^2+^/Pb^2+^ translocation, respectively^5^. It is generally accepted that P_1B_-type ATPases have high specificity for the heavy-metal ions they transport. The substrate specificity is presumably relies on the conserved residues in transmembrane segments H6, H7, and H8, but remains to be established^6^. Another important structure of P_1B_-ATPases is the presence of cytoplasmic N-MBDs (N-terminal metal binding domains). Current studies mainly focus on the N-MBDs featuring prototypical GXXCXXC motif(s). The N-MBDs appear to be responsible for sensing cellular metal ions and regulating the ATPase enzyme activity as well as conferring ionic specificity^7^,^8^. In contrast, much less is known about the role of N-MBDs containing His-rich motif which is mainly present in the P_1B2_- and P_1B3_-type ATPase^6^.

In most Gram-negative bacteria, the expression of Cu ATPase is mainly regulated by transcriptional activator CueR; whereas Gram-positive bacterial Cu ATPase is repressed by CsoR or CopY^6^. CsoR was initially identified in *M. tuberculosis*, and subsequently in other Gram-positive bacteria such as *Bacillus subtilis*, *Corynebacterium glutamicum*, *Listeria monocytogenes*, and *Streptomyces lividans*^6^. Experimental evidence for Cu homeostasis gene regulation by CsoR in Gram-negative bacteria is still lacking to date. *Thermus thermophilus* CsoR is the only instance identified in Gram-negative bacteria, however, metal-binding motif of the CsoR (H-C-H-H) is the same as that of *E. coli* RcnR, which is distinctly different from that of CsoRs (C-H-C) from Gram-positive bacteria above^9^.

Rhizobia are Gram-negative soil-dwelling bacteria that form a symbiosis with legumes to fix nitrogen from the atmosphere^10^. Recently, the nitrogen fixer has attracted great attention for their role in aiding phytoremediation of metal contaminated soils^11^,^12^. Cu, a ubiquitous transition metal, enters soils via agricultural and industrial activities, and exposure at high levels have presented serious threats to the environment and human health^13^. Some rhizobia can tolerate high concentrations of Cu and display the potential phytoremediation by their host plants in Cu contaminated soil^11^,^12^,^14^,^15^. However, Cu resistance determinants of rhizobia are poorly characterized.

In the present study, the mechanisms of Cu resistance in *Bradyrhizobium liaoningense* CCNWSX0360 were investigated through random transposon mutagenesis. A *csoR*-*cueA* divergon encoding a CsoR-like repressor and a heavy metal transporting P-type ATPase (CueA) was functionally characterized; *csoR*-*cueA* divergon plays a crucial role in Cu homeostasis, and also involves in Zn/Cd resistance suggesting a versatile metal resistance component. Furthermore, the role of *cueA* in symbiotic nodulation under Cu stress was investigated, which will contribute to improving the metal bioremediation potential of legume-rhizobium symbiosis.

## Results

### Isolation and phylogenetic identification of the Cu resistant isolate Bln0360

A total of 108 rhizobia were isolated from the nodules of 13 leguminous plant species in the study. Among them, strain CCNWSX0360 from *Vigna unguiculata* showed the highest resistance to Cu (2.0 mM) and was selected for the study. The 16S rRNA gene sequence of strain CCNWSX0360 (KU507314) showed 100% similarity to *B*. *liaoningense* SEMIA 5022 (FJ390920) and 99.9% similarity to strain 2281^T^ (AJ250813). Phylogenetic analysis revealed that strain CCNWSX0360 belonged to *B. liaoningense* (Fig. S1) and it was named *B. liaoningense* CCNWSX0360 (Bln0360). The maximum tolerable metal concentrations (MTCs) of Bln0360 to the test metals were 2.0, 3.2, 0.15, 2.8, 0.6, 1.4, and 0.1 mM for Cu^2+^, Zn^2+^, Cd^2+^, Pb^2+^, Ni^2+^, Co^2+^, and Ag^+^ (Table 1).

### Identification of genes involved in Cu resistance by transposon mutagenesis

To identify genes involved in Cu resistance in Bln0360, a transposon mutant library (17,247 Tn5-insertions) was constructed. Upon screening, six Cu sensitive mutants were obtained (Table 1). To further test the sensitivity and specificity, the MTCs of various metal ions (Cu^2+^, Zn^2+^, Pb^2+^, Cd^2+^, Co^2+^, Ni^2+^, and Ag^+^) for each mutant were determined in TY (Tryptone-Yeast) medium. Of the mutants, three mutants (Bln-c, Bln-54, and Bln-29) exhibited a drastic reduction in Cu tolerance, with MTCs much lower (0.6 mM Cu^2+^) than that of Bln0360 (2.0 mM Cu^2+^). Bln-29, Bln-32 and Bln-54 also showed the varying of decreased tolerance toward other metals ions (Zn^2+^, Pb^2+^, Cd^2+^, Co^2+^, and Ni^2+^), but did not exclusively affect the resistance to Cu^2+^. In contrast, no difference in tolerance to metals other than Cu^2+^ was observed between the mutants (Bln-c, Bln-163, and Bln-d) and the wild-type strain.

**Figure 1:**
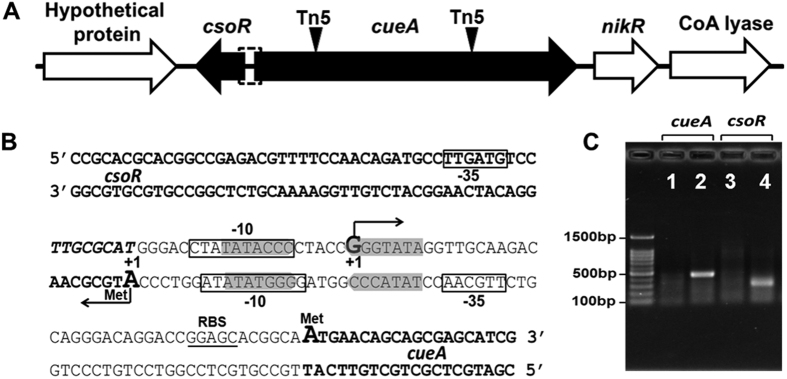
Organization of the *csoR-cueA* divergon and their promoter regions from Bln0360. **(A)** Schematic representation of the arrangement of the *csoR*-*cueA* divergon; the positions of Tn*5* insertions are indicated by inverted black triangles. **(B)** Nucleotide sequences of the bidirectional *csoR*-*cueA* promoter region. Partial coding sequences for *csoR* and *cueA* are indicated in bold. The transcriptional start sites (+1) of *csoR* and *cueA* are indicated by large bold letter, and the ribosome binding site (RBS) of *cueA* is underlined. The predicted −35 and −10 elements of the *csoR* and *cueA* promoter are boxed. The 19-bp putative CsoR-box with a 7-bp inverted repeat is indicated with a grey background. **(C)** RACE experiments were performed using RNA isolated from uninduced (lanes 1 and 3) or 0.8 mM CuSO_4_-induced (lanes 2 and 4) cultures of Bln0360.

Among these mutants, Tn*5* was inserted into the same gene encoding a putative heavy metal-transporting P-type ATPase, named *cueA* (KU665989), in both strains Bln-d and Bln-163, which was consistent with their identical tolerance to the tested metals in this study (Fig. 1A and Table 1). In strain Bln-32, the interrupted gene *tolC* (KU665993) encoded an outer membrane protein showing 21% identity to *E. coli* TolC, which is an outer membrane component of a multidrug efflux system, AcrAB-TolC^16^. In Bln-c, the interrupted gene *copA* (KU665990) encoded a multicopper oxidase showing 98% identity to CopA from *Bradyrhizobium diazoefficiens* USDA 110^17^. Moreover, individual genes *lptE* (KU665991) and *ctpA* (KU665992) encoding putative membrane formation associated proteins were respectively interrupted in Bln-29 and Bln-54^18^,^19^.

Further analysis of the sequences derived from Bln-163 and Bln-d identified a small open reading frame, named *csoR* (KU665989), which was inversely oriented and located immediately upstream of *cueA* (Fig. 1A). The ORF encodes a putative CsoR-like regulator which has mainly been reported in Gram-positive bacteria^6^. The remainder of the experiment focuses on the genetic arrangement and function of the *cueA*-*csoR* divergon from the Gram-negative bacteria.

### *cueA* is critical for Cu resistance of Bln0360

*In silico* analysis showed that *cueA* encodes a putative protein of 815 amino acid residues with a theoretical molecular mass of 85.5 kDa. The deduced CueA amino acid sequence showed high identity with several previously characterized Cu transporting P-type ATPases: CopA of *M. amorphae* (EHH02252, 58.5%)^11^, CopA of *A. tumefaciens* (Atu0937, 42.3%)^20^ and CopA of *E. coli* (BAE76263, 38.2%)^21^. Alignment of sequences revealed three conserved domains of the P-type ATPase family, including a SGES phosphatase domain (A-domain), a DKTGT aspartyl kinase domain (P-domain), and a GXGXND ATP-binding domain (N-domain), which were also present in CueA (Fig. S2). CueA contained eight predicted transmembrane segments (TMS), with the CPX motif (CPC) located in TMS6 and the signature sequences NY in TMS7 and MXXSS in TMS8. These *in silico* data allowed assignment of CueA to subclass type P_1B1_ according to Palmgren’s classification^5^.

**Figure 2:**
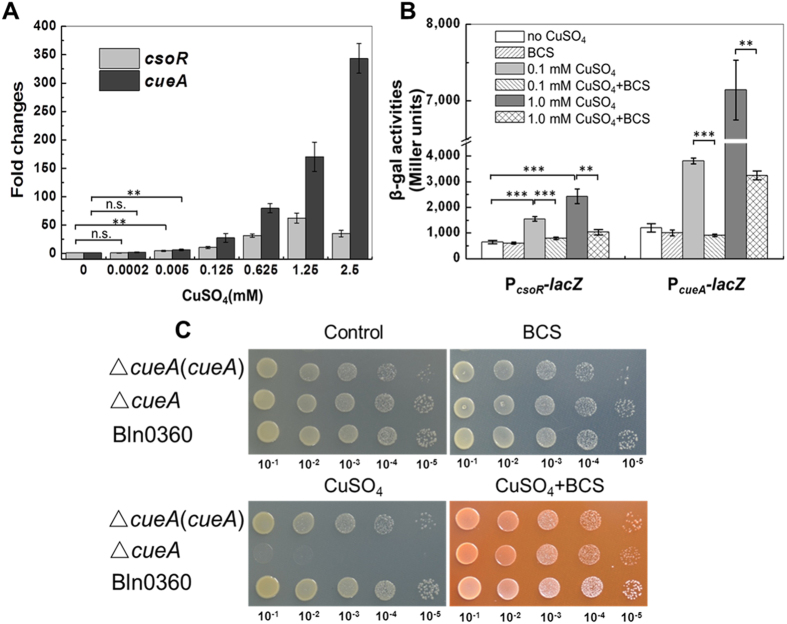
c*ueA* contributes to Cu resistance in Bln0360. **(A)** Transcription of *cueA* and *csoR* in response to Cu^2+^. The cDNA was derived from Bln0360 culture at mid-log phase after induction for 2 h with elevated concentrations of CuSO_4_. Normalized expression of *cueA* and *csoR* with respect to 16S rRNA expression is presented as the mean ± SD (standard deviation) of data from three independent samples. Relative mRNA levels were expressed as fold change with respect to the untreated control. **P < 0.01. **(B)** β-Galactosidase activity of P_*csoR*_::*lacZ* and P_*cueA*_::*lacZ* reporters were determined in TY medium at 0.1 mM or 1.0 mM CuSO_4_ with and without the addition of 1.0 mM bathocuproine disulfonate (BCS). Values are presented as the mean ± SD of data from three independent experiments. ***P < 0.001; **P < 0.01. **(C)** Tolerance levels of Bln0360, the Δ*cueA* mutant and the Δ*csoR*(*csoR*) complemented strain to Cu^2+^. Ten-fold serial dilutions of log phase culture were spotted onto TY agar plates with no addition, 1.0 mM BCS, 0.8 mM CuSO_4_ or 0.8 mM CuSO_4_ + 1.0 mM BCS. Plates were photographed after 7 d incubation at 28 °C.

qRT-RCR analysis of the Cu^2+^ responsiveness of *cueA* showed that there was a gradual response with elevated concentration of added CuSO_4_ (Fig. 2A), suggesting a significant dose-dependent effect. A notable induction was observed when 0.005 mM CuSO_4_ was added to cultures (*P* < 0.01). Induction of *cueA* reached up to ~350-fold relative to untreated cells, at 2.5 mM (Fig. 2A), though this was beyond the maximum Cu^2+^ concentration the bacteria could tolerate in TY plate. Dose-dependent induction of *csoR* by CuSO_4_ also began at 0.005 mM, but reached a maximum at 1.25 mM (Fig. 2A). Addition of the Cu^+^-specific chelating agent bathocuproine disulfonate (BCS, 1.0 mM) to the cultures completely eliminated the induction caused by 0.1 mM Cu^2+^, and partially eliminated that caused by 1.0 mM Cu^2+^ (Fig. 2B). These results suggested that expression of *cueA* could be induced by Cu^+^. Although Cu^2+^ was added to the medium, it would be reduced to Cu^+^, intracellularly; therefore, Cu^+^ might be the actual inducer^21^,^22^.

To verify the Cu sensitive phenotype of Tn*5* insertions Bln-d and Bln-163 was indeed caused by inactivation of *cueA*, an in-frame deletion mutant (Δ*cueA*) of Bln0360 was constructed and subjected to metal tolerance assays. No difference was observed in growth under various metal stresses between the transposon and constructed mutants (data not shown), indicating that no polar mutations were produced by insertion of Tn*5*. Complementation of the Δ*cueA* mutant with full-length *cueA* gene restored the Cu resistance to near that of the wild-type (Fig. 2C).

The Cu sensitivity of Δ*cueA* on TY agar medium containing 0.8 mM CuSO_4_ was completely eliminated by adding 1.0 mM BCS (Fig. 2C). Thus, Cu toxicity to Bln0360 may be dependent on the conversion of Cu^2+^ to Cu^+^, and also implies that CueA plays a role in protection of Bln0360 from Cu^+^, which agreed with the responses of the Cu^2+^-inducible expression of *cueA* to BCS (Fig. 2B). Overall, these data demonstrated that CueA is involved in and plays the major role in intracellular Cu detoxification via Cu^+^ efflux.

### Two putative N-terminal MBDs is required for full function of CueA

**Figure 3:**
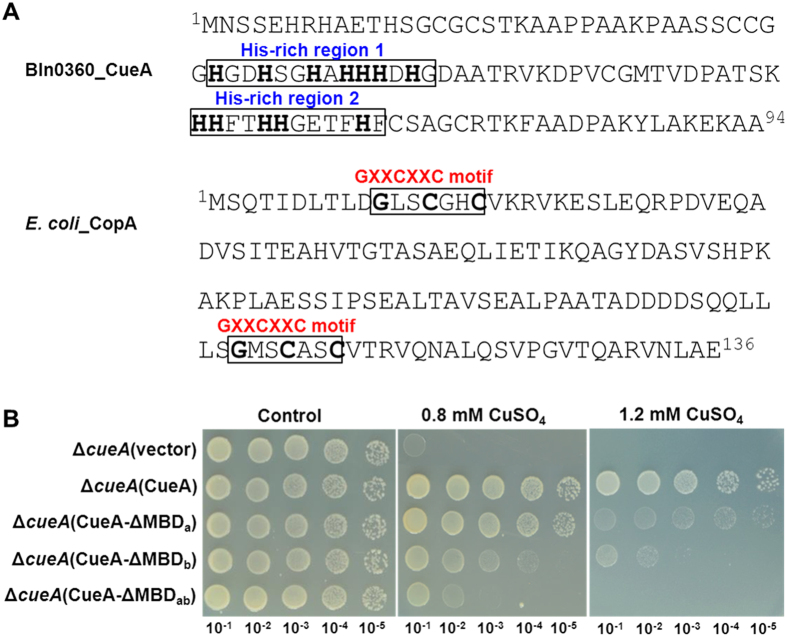
The N-terminal His-rich stretchs are required for full Cu resistance of CueA. **(A)** Characteristic metal binding motifs in the N-terminus of CopA from *E. coli* and CueA from Bln0360. His-rich stretch and conserved residues within GXXCXXC motif are highlighted. Superscript numbers represent amino acid positions. **(B)** Comparative Cu tolerance of Δ*cueA* strains expressing CueA and its variants on TY agar plates containing 0.8 or 1.2 mM CuSO_4_. Plates were photographed after 7 d incubation at 28 °C. Strain Δ*cueA*(vector) was used as the negative control. MBD_a_ indicates deletion of the first His-rich stretch, MBD_b_ indicates deletion of the second, and MBD_ab_ indicates deletion of both.

Unlike other P_1B1_-type ATPases that possess the typical N-terminal GXXCXXC motif, CueA contains two His-rich stretchs (Fig. 3A). The presence of His-rich stretch in the CueA is unusual, in that P_1B_-type ATPase with His-rich N-MBDs are usually involved in divalent metal ions transportation^23^,^24^. To explore the role of the two putative MBDs in the Cu resistance mediated by CueA, three variants CueA-ΔMBD_a_ (with the first His-rich stretch deleted), CueA-ΔMBD_b_ (with the second His-rich stretch deleted) and CueA-ΔMBD_ab_ (with both His-rich stretchs deleted) were expressed in the Cu sensitive Δ*cueA* mutant. Tolerance assay of these transformants indicated that deletion of the MBD_b_ largely abrogated the Cu resistance mediated by CueA, whereas observable reduction caused by deletion of the MBD_a_ only observed on 1.2 mM CuSO_4_-supplemented plates (Fig. 3B). The result indicated that both the putative His-rich domains were obligatory for the function of CueA and furthermore the second domain showed a dominant role. Note that the Δ*cueA* mutant expressing CueA-ΔMBD_ab_ had a distinct growth advantage compared to the mutant with empty plasmid at Cu^2+^ concentrations of 0.8 mM. It indicated that CueA is capable of functioning in a manner independent of the N-terminal MBDs (Fig. 3B). These data support the model in which N-terminal MBDs is responsible for regulation rather than an absolutely essential for the catalytic mechanism of P_1B_-ATPase^25^.

### CsoR negatively regulates the *csoR*-*cueA* divergon

*csoR* encodes a putative protein of 91 amino acid residues showing 44.4% and 27.6% identities with CsoR from *C. glutamicum* (AIK84123) and *M. tuberculosis* (P9WP49), respectively^26^,^27^. Alignment of the deduced protein with its orthologs revealed the presence of a conserved C-H-C motif (Cys^33^, His^58^ and Cys^62^), which served as Cu^+^-binding ligands (Fig. S4), furthermore, I-TASSER software predicted three alpha helices similar to the *M. tuberculosis* CsoR, suggesting a similar mechanism to sense and respond to Cu^+^.

Inspection of the intergenic region between *csoR* and *cueA* identified −10 and −35 promoter sequences separately in the upstream region of each gene; the −10 elements partially overlapped (Fig. 1B). The transcription start site (TSS; position +1) of *cueA* was mapped to the G nucleotide located 42 nucleotides upstream of the putative start codon (ATG) by 5′ rapid amplification of cDNA ends (RACE) (Fig. 1B,C). Interestingly, the putative TSS of *csoR* was mapped to the first nucleotide (A) of start codon of this gene, suggesting a leaderless *csoR* mRNA, in which the TSS is starts directly with a 5′-terminal AUG^28^. Further examination of the sequence upstream of the TSS revealed a 7-bp inverted repeat separated by 5 bp (5′-TATACCCCTACCGGGTATA-3′), which highly similar to the recognition motif (CsoR-box) of the CsoR from *C. glutamicum*^27^. The CsoR-box overlapped with the overlapping −10 element of *cueA* and *csoR*, indicating a bidirectional promoter structure. The conserved sequence motif of CsoR and the location of the CsoR-box suggest that it regulates expression of both transcripts (i.e., *csoR* and *cueA*) simultaneously, in opposite directions.

**Figure 4:**
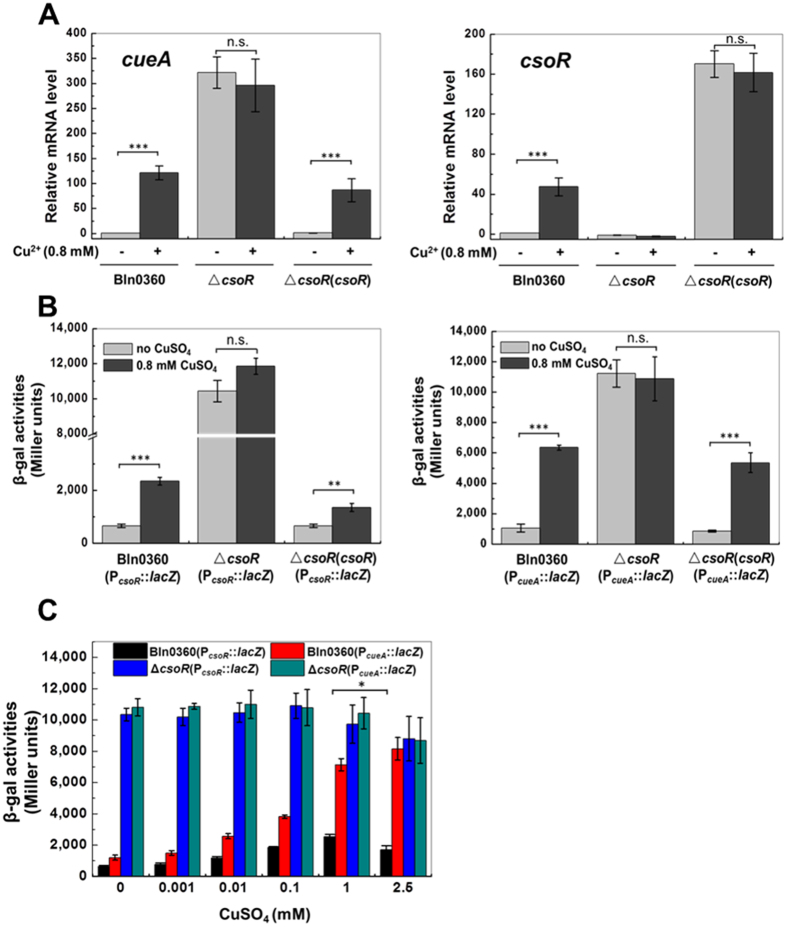
Effects of *csoR* deletion on the expression of the *csoR-cueA* divergon and the tolerance of Bln0360 to Cu exposure. **(A)** The relative mRNAs levels of *cueA* (left) and *csoR* (right) in Bln0360, the Δ*csoR* mutant and the Δ*csoR*(*csoR*) complemented strain exposed to media containing or lacking 0.8 mM CuSO_4_ for 2 h. mRNA levels are presented relative to the untreated wild-type strain. **(B)** β-Galactosidase activity of P_*csoR*_::*lacZ* (left) and P_*cueA*_::*lacZ* reporter (right) in wild-type Bln0360, the Δ*csoR* mutant and the Δ*csoR*(*csoR*) complemented strain grown in TY medium with and without the addition of 0.8 mM CuSO_4_ for 2 h. **(C)** Expression of the *csoR*-*cueA* divergon in wild-type Bln0360 and Δ*csoR* mutant in response to elevated concentrations of CuSO_4_. The SD from three independent experiments is indicated on each bar. *P < 0.05, **P < 0.01, ***P < 0.001.

To elucidate whether or not the *csoR-cueA* divergon is autoregulated by CsoR, the transcription of *csoR* and *cueA* in response to Cu^2+^ were examined by Quantitative real-time PCR (qRT-PCR) in the Δ*csoR* mutant and the Δ*csoR*(*csoR*) complemented strain (Fig. 4A,B). In the Δ*csoR* mutant, a high level of *csoR* and *cueA* mRNA was detected even without the addition of CuSO_4_, indicating uncontrolled transcription. Complementation of the mutant with *csoR* gene resulted in high level transcription of *csoR* and restored the Cu^2+^-dependent induction of *cueA*, suggesting that *csoR* acts as a negative regulatory factor.

The β-galactosidase activity of P_*csoR*_::l*acZ* and P_*cueA*_::*lacZ* fusions was determined to further confirm the data of the two genes expression. As shown in Fig. 4B,C, expression of P*csoR*::*lacZ* and P*cueA*::*lacZ* was completely derepressed in the *csoR* mutant, which was consistent with the results of qRT-PCR. Complementation of the mutant restored the repression and Cu^2+^-responsiveness of P_*csoR*_::*lacZ* and P_*cueA*_::*lacZ* (Fig. 4B). The results provided further evidence that CsoR negatively regulates the *csoR*-*cueA* divergon. It is noteworthy that fusion reporter P_*cueA*_::*lacZ* expressed 1.9–4.8 fold higher β-galactosidase activity than the P_*csoR*_::*lacZ* fusion at each Cu^2+^ concentration in the wild-type strain (Fig. 4C). In contrast, in the CsoR deletion mutant, the β-galactosidase activity of each fusion was indistinguishable, indicating parallel promoter strength. These data showed that CsoR controlled the promoters of both *csoR* and *cueA* simultaneously, but influenced their activity to varying extents. Notably, the expression level of *csoR* decreased when the induction concentration of Cu^2+^ was 2.5 mM as seen from its transcript abundance relative to that at lower Cu^2+^ concentrations (Figs 4C and 2A), which is probably a result of competition between RNA polymerases for the respective promoters. Collectively, these data clearly showed that CsoR acts as a repressor to regulate the *csoR*-*cueA* divergon expression via a bidirectional promoter.

### Expression of the *csoR-cueA* divergon could be induced by Zn^2+^ and Cd^2+^

**Figure 5:**
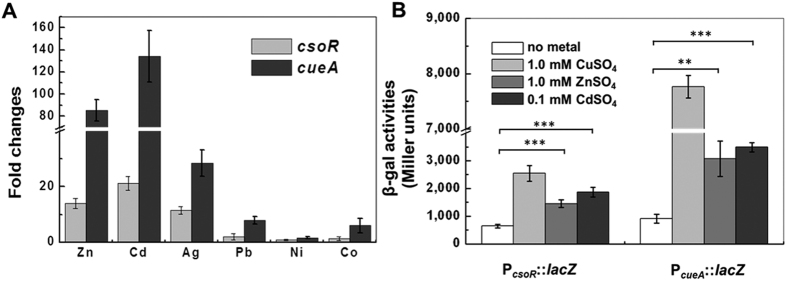
The *csoR*-*cueA* divergon is up-regulated by Zn^2+^ and Cd^2+^. **(A)** mRNA expression levels of *csoR* and *cueA* genes in response to different metal ions. cDNA was derived from mid-log phased Bln0360 culture after 2 h incubation with 1.0 mM CuSO_4_, 0.1 mM CdSO_4_, 0.075 mM AgNO_3_, 2.0 mM PbNO_3_, 0.4 mM NiSO_4_ or 1.0 mM CoCl_2_ (bacterial growth was markedly inhibited by the above specified concentrations of metal ions) and subjected to qRT-PCR assay. Fold change of gene expressions was estimated mRNA levels compared to the untreated cultures. **(B)** Expression of the *csoR*-*cueA* divergon in response to Cu^2+^, Zn^2+^, and Cd^2+^. Strain Bln0360 carrying P_*csoR*_::*lacZ* or P_*cueA*_::*lacZ* fusions were grown in TY medium to log phase and individually supplemented with 1.0 mM CuSO_4_, 1.0 mM ZnSO_4_ or 0.1 mM CdSO_4_. Cells were collected and β-Galactosidase activities were determined. Error bars indicate SD of triplicates. ***P < 0.001; **P < 0.01.

Since CsoR in some bacteria (e.g., *T. thermophilus* and *C. glutamicum*) can sense various metal ions and derepress transcription^9^,^27^, we investigated whether metal ions other than Cu^2+^ could induce expression of the *csoR*-*cueA* divergon in Bln0360. The result showed that the mRNA expression of *cueA* gene could be markedly induced by Zn^2+^ and Cd^2+^ (~90-fold for 1.0 mM Zn^2+^ and ~135-fold for 0.1 mM Cd^2+^, respectively) as well as Cu^2+^. Similar up-regulation of *csoR* was also observed, but with lower fold-changes (Fig. 5A).

To further confirm the expression pattern of the divergon by Zn^2+^ and Cd^2+^, we determined β-galactosidase activity of P_*csoR*_::*lacZ* and P_*cueA*_::*lacZ* in response to the two metal ions. As Fig. 5B shows, 1.0 mM Zn^2+^ caused a 3.4-fold (*P* < 0.01) and 0.1 mM Cd^2+^ caused a 3.8-fold increase (*P* < 0.01) in the expression of *cueA* compared with the treatment in the absence of metal ions. Significant increases (1.9-fold for Zn^2+^ and 2.7-fold for Cd^2+^) were also observed in the expression of *csoR* in response to the same concentration of Zn^2+^ and Cd^2+^. Additionally, expressions of *csoR* and *cueA* were markedly higher in the treatment of 1.0 mM Cu^2+^ than that in the same concentration of Zn^2+^ (Fig. 5B). Combined with the data of responses to BCS above, these results indicated that *csoR*-*cueA* divergon can be induced by both monovalent Cu^+^ and divalent Zn^2+^/Cd^2+^ and may be involved in detoxification of multiple heavy metals.

### *csoR* mutation increase resistance to Cu, Zn and Cd

**Figure 6:**
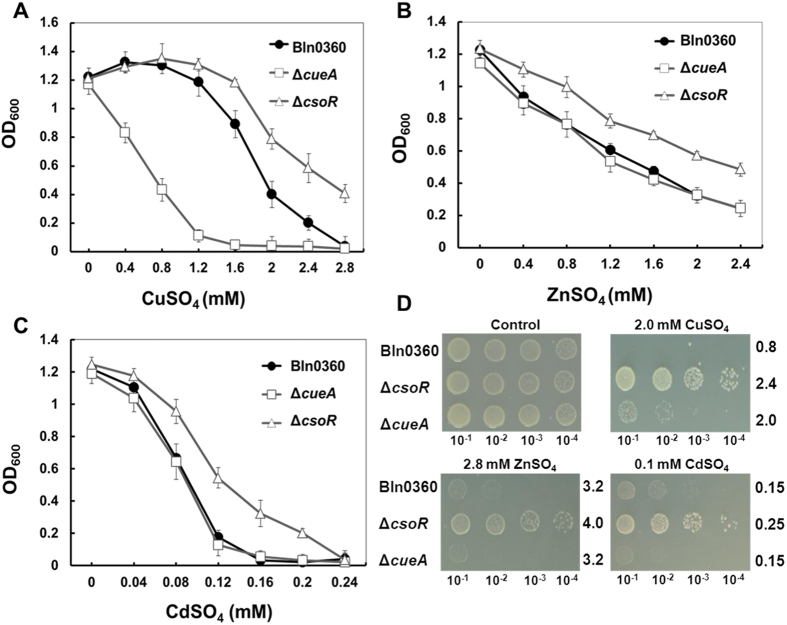
Deletion of the *csoR* gene from Bln0360 increased tolerance of strain to Cu, Zn and Cd. Growth curves of wild-type Bln0360, Δ*csoR* and Δ*cueA* strain in TY liquid media exposed to different levels of CuSO_4_ **(A)**, ZnSO_4_ **(B)**, and CdSO_4_ **(C)**. Samples were taken and the optical densities (600 nm) were determined when the wild type in the absence of metal ions reached the stationary phase (7 d). Error bars are SD of triplicates. **(D)** Comparative Cu/Zn/Cd tolerance between Bln0360, Δ*csoR* and Δ*cueA* strain on TY agar medium containing 2.0 mM CuSO_4_, 2.8 mM ZnSO_4_ or 0.1 mM CdSO_4_. The MTC values are depicted at the right.

The *csoR*-deficient mutant was selected to test the altered tolerance to Cu, Zn, Cd, Ni and Ag, respectively. Since CueA was evidenced to facilitate Cu tolerance, the strain deficient in its repressor CsoR should be more resistant to Cu as compared to wild-type strain. As expected, Δ*csoR* mutant displayed a distinct growth advantage in the media containing high concentration (>1.6 mM) of Cu^2+^ compared to Bln0360, as determined by culture optical density and plate assay (Fig. 6A,D); the MTC of Cu^2+^ for Δ*csoR* mutant was raised from original 2.0 to 2.4 mM (Fig. 6D).

Intriguingly, deletion of the *csoR* gene also increased tolerance of strain to Zn and Cd, though mutation of its target gene (*cueA*) had no measurable impact on the tolerance to these two metals. As shown in Fig. 6B, Δ*csoR* mutant showed markedly higher OD_600_s than Bln0360 in ZnSO_4_-supplemented TY broth. Similarly, Bln0360 showed a sharp reduction in growth after 0.08 mM CdSO_4_, approaching zero growth at approximately 0.16 mM (Fig. 6C); in contrast, Δ*csoR* mutant remains tolerant until approximately 0.2 mM. The hyper-tolerant phenotype was also observed by plate assay and the MTCs of ZnSO_4_ and CdSO_4_ for Δ*csoR* mutant were 4.0 mM and 0.25 mM, respectively, which were higher than 3.2 mM and 0.15 mM for wild-type (Fig. 6D and Table 1). We speculated that one possible reason could be due to the depression of general stress genes in the transcript level, but such increased tolerance was not observed when Δ*csoR* mutant was tested on both solid and liquid medium supplemented with Ni^2+^ and Ag^+^ (data not shown).

### *cueA* confers resistance to Zn and Cd

**Figure 7:**
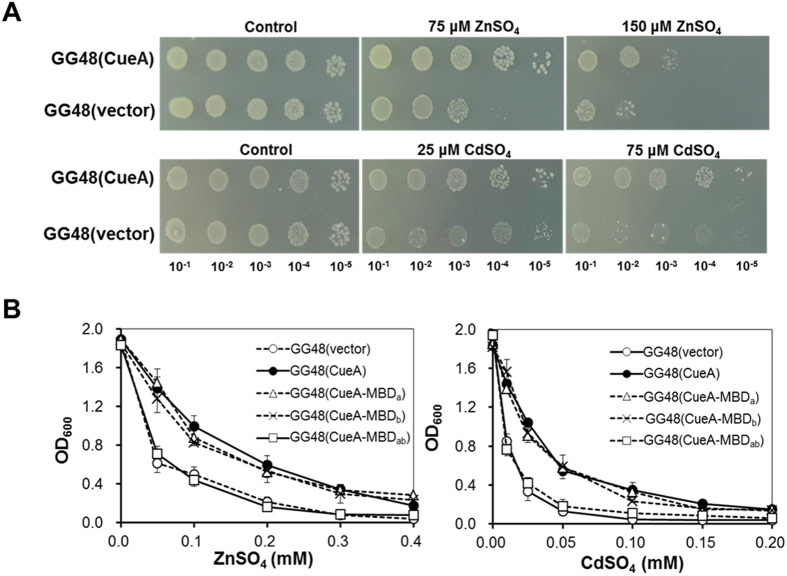
CueA is involved in Zn and Cd resistance. **(A)** Zn and Cd resistance of *E*. *coli* GG48 expressing CueA. *E. coli* GG48 with empty plasmid was used as negative controls. **(B)** Effects of N-terminal His-rich stretch deletion on the function of CueA in Zn/Cd resistance. Growth of *E*. *coli* GG48 expressing different truncated versions of CueA was compared in LB medium containing various concentrations of ZnSO_4_ (left) or CdSO_4_ (right). Symbols represent *E*. *coli* GG48 expressing empty plasmid, full-length CueA, CueA-ΔMBD_a_, CueA-ΔMBD_b_ and CueA-ΔMBD_ab_) (○, ●, △, ×, and □, respectively).

Since Δ*csoR* mutant displayed an increased tolerance to Zn/Cd and *cueA* expression was up-regulated by the two metals, we hypothesized that *cueA* might contribute to Zn and Cd resistance. To validate this hypothesis, construct pBBR5-*cueA* was introduced into the Zn/Cd sensitive *E. coli* GG48, and the growth of resultant strain was monitored on Luria-Bertani^19^ medium containing different concentrations of CdSO_4_ or ZnSO_4_. Spot assays clearly indicated that *E. coli* GG48 expressing CueA had a distinct growth advantage on both ZnSO_4_- and CdSO_4_-supplemented agar plates when compared with the *E. coli* harboring the empty plasmid (Fig. 7A). For Cd, the *E. coli* bearing empty plasmid barely grew on the plate containing CdSO_4_, whereas cells with CueA expression plasmids appeared relatively robust growth (Fig. 7A). Consistent results were obtained when growing in the liquid media (Fig. 7B). Thus, the Zn/Cd sensitive *E. coli* strain appeared to use CueA as a resistance enhancer.

To test whether the Zn/Cd resistance mediated by CueA is related to the unusual N-terminus, three CueA variants, CueA-ΔMBD_a_, CueA-ΔMBD_b_ and CueA-ΔMBD_ab_, were expressed in *E. coli* GG48 and the cells were subjected to Zn/Cd tolerance assays. As Fig. 7B shows, no significant growth difference was observed in the LB media containing indicated concentration of ZnSO_4_ or CdSO_4_ between *E. coli* GG48 (CueA) and the variants *E. coli* GG48 (CueA-ΔMBD_a_) or *E. coli* GG48 (CueA-ΔMBD_b_). However, the growth of the *E. coli* GG48 (CueA-ΔMBD_ab_) was decreased to the same extent as that of *E. coli* GG48 (vector), when both MBDs were excised. These results suggested that the two N-terminal MBDs were essential for the Zn and Cd resistance enhancement associated with CueA mediation.

### CueA is required for plant colonization in Cu overloaded conditions

**Figure 8:**
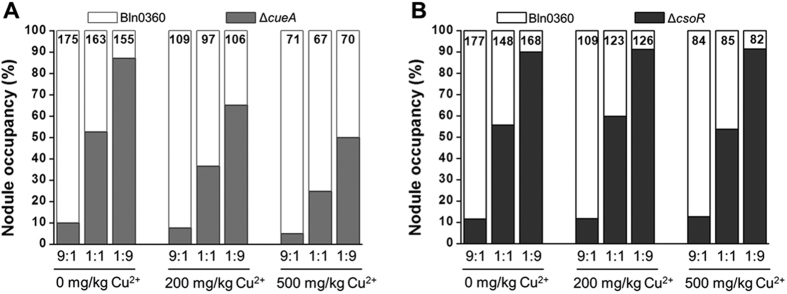
Effects of Cu resistance on competition for plant nodulation by Bln0360. The Δ*csoR* mutant **(A)** or Δ*cueA* mutant **(B)** was mixed with Bln0360 in indicated ratios (1:9, 1:1 or 9:1) and applied to *V. unguiculata* seedlings. Significant differences between the expected and observed colonization percentages were evaluated by chi-square test at a confidence level of 0.05. The statistics did not include nodules containing both strains. The number indicated on each bar represents the total number of nodules from nine plants.

Considering that Cu resistance could protect the strain Bln0360 against excess Cu-induced damage, thus, we speculate that loss of the Cu pump would affect the nodulation performance of the strain in such conditions. Hence, the wild-type strain and one of each single mutant were combined at three different ratios and then applied to plants. As Fig. 8A shows, the Δ*cueA* mutant displayed a lower competitiveness than Bln0360 in Cu-supplemented conditions, as the observed proportion of nodule occupancy by the Δ*cueA* mutant was significantly (*P* < 0.05) lower than the expected proportion at every inoculum ratio. At a ratio of 1:1, the percentages of the Δ*cueA* mutant recovered from nodules were 36.47% and 24.77% in the presence of 200 mg/kg and 500 mg/kg CuSO_4_, respectively (Fig. 8A); no distinct effects on competitiveness were observed when plants were grown without Cu treatment. In contrast, the Δ*csoR* mutant was equally competitive with the wild-type strain in nodule occupancy in all treatments (Fig. 8B). Collectively, these results show that CueA is required for the nodulation performance of Bln0360 in Cu rich conditions.

## Disscussion

Analysis of mutants generated by Tn*5* revealed that at least three strategies were adopted to alleviate Cu toxicity in Bln0360 (Table 1). Mutants Bln-d and Bln-163 with P_1B1_-type ATPase CueA-insertion displayed a specific sensitivity to Cu showing the critical role of this transporter in Cu resistance. In Bln-32, a TolC family protein encoded by *tolC* may be involved in Cu transportation; TolC in *E.coli* is an outer membrane components of type I secretion system that can extrude noxious agents such as antibiotics and toxic metal ions. In addition to the efflux-mediated mechanism, Bln0360 adopts enzymatic detoxification against superfluous Cu, as mutant Bln-c with multicopper oxidase encoding gene *copA*-interruption was extremely sensitive to Cu; multicopper oxidase was thought to protect the periplasm from Cu-induced damage through oxidization of Cu^+^ to less toxic Cu^2+^ in aerobic conditions^29^. Moreover, two membrane integrity related genes (*ctpA* and *lptE*) presumably play a house-keeping role in protecting Bln0360 cells from Cu toxicity, via their function in maintaining the protective permeability barrier of the cell^18^,^19^,^30^.

Homology and transmembrane signature sequences assigned CueA into group P_1B1_ type (Fig. S2)^25^. ATPases in this group carry out the function of intracellular Cu^+^/Ag^+^ detoxification through pumping Cu^+^/Ag^+^ from the cytoplasm into the periplasm^7^,^21^,^31^. Mutation and complementation analysis showed that *cue*A to play a critical role in Cu detoxification in Bln0360 (Fig. 2C). Given that the toxicity caused by adding CuSO_4_ could be eliminated by adding the Cu^+^-specific chelator BCS (Fig. 2C), it was concluded that CueA could carry out the function of Cu^+^ efflux. It should be note that no altered Ag^+^ tolerance by deletion of *cueA* or *csoR* had been observed, indicating that Ag^+^ may be not the substrate of the transporter.

It is accepted that there are few or no “free” (bioavailable) Cu ions in the bacterial cell^32^; therefore, strict regulation of Cu homeostasis is very critical. Expression of CueA was negatively regulated by CsoR repressor, similar to the regulation of Cu transporting P-type ATPase in Gram-positive bacteria^6^. In phylogenetically related *A. tumefaciens*, however, expression of CopA was positively regulated by a CueR-like activator, similar to that in *E. coli*^6^. A BLASTN search of the sequence of *csoR-cueA* divergon against the sequenced *Bradyrhizobium* genomes found that the organization genetically linking CsoR repressor and Cu ATPase is conserved, suggesting a primary regulator architecture in this genus (unpublished data from our lab). Under Cu limited condition, CsoR represses transcription via binding to the operator-promoter region of the target gene; upon binding Cu^+^, CsoR is released to form the CsoR-DNA complex, resulting in transcription occurred^33^. In our study, *csoR* and *cueA* were highly expressed in the *csoR*-deleted mutant (Fig. 4A–C), which logically was due to the exposed operator-promoter region allowing the access of the RNA polymerase. The promoters of *csoR* and *cueA* had parallel strength in the CsoR-deficient background; however, the expression levels of *cueA* were far higher than those of *csoR* in the wild-type strain (Fig. 4C). That could be due to the presence of bidirectional promoter and the CsoR-box located in the overlapped promoters of *cueA* and *csoR* (Fig. 1C). As a result, coordination/competition between the RNA polymerase and the CsoR constitutes a significant part of the regulatory process^34^. In other cases, the two promoters occupied the same sequence element on opposing DNA strand, therefore, the collision between RNA polymerases likely influenced the expression of each gene. In *csoR*-*cueA* divergon, the expression of CsoR repressor was regulated by itself. Coordination between the expression and derepression of transcriptional repressor allows the regulation process under a more narrow control^35^.

Studies have shown that Cu^+^, but not other metal ions, bind to the CsoR of *M. tuberculosis* and *L. monocytogenes*, thereby relieving the interaction between this regulator and promoter DNA and allowing transcription to proceed^26^,^36^. However, expression of the *csoR*-*cueA* divergon from Bln0360 could be induced by Cu^2+^ as well as by Zn^2+^ and Cd^2+^ (Fig. 5A). The Zn^2+^-inducible expression of *csoR* regulons has been reported in *C. glutamicum*^27^,^37^. For Cd^2+^-inducible expression, it is easy to understand in that Zn^2+^ and Cd^2+^ share similar coordination geometries^38^. According to the derepression mechanism, the effects of these metal ions binding between CsoR and its operator-promoter DNA was reflected in the expression of the *csoR-cueA* divergon. Our findings confirmed that the role of metal ions (Cu^2+^, Zn^2+^ and Cd^2+^) in the inhibition of DNA binding activity (Fig. 5B). We also find Zn^2+^-induced expression of *csoR-cueA* divergon was less than Cu^2+^ or Cu^+^ (Figs 2A and 5), in agreement with previous study in *C. glutamicum*^27^. Additionally, the CsoR-box (5′-TATACCCnnnnnGGGTATA-3′) of *B. liaoningense* showed higher similarity to *C. glutamicum* (5′-ATACCCCnnnGGGGTAT-3′), suggesting a similar coordination property of metal cations by the two CsoRs. *T. thermophilus* CsoR can coordinate multiple metal ions including divalent Cu^2+^/Zn^2+^/Cd^2+^ and monovalent Cu^+9^. However, the metal-binding motif of *T. thermophilus* CsoR is H-C-H-H, instead of specific C-H-C being conserved in known CsoRs from other bacteria^9^. Sakamoto K *et al*. thought that the low selectivity of *T. thermophilus* CsoR to metal ions is due to the presence of His(70) and His(5) residues^9^. Likewise, we did not find the additional His residue in Bln0360 CsoR corresponding to the His(5) residue in *T. thermophilus* CsoR. Therefore, further studies need to elucidate the critical role of *B. liaoningense* CsoR in the Zn^2+^- and Cd^2+^-dependent expression of *csoR-cueA* divergon.

In this study, expression of *cueA* was found to be up-regulated by Cu^2+^/Zn^2+^/Cd^2+^ stress (Figs 2 and 5), suggesting that *cueA* was involved in protecting cells against these metals toxicity. This is validated by complementation studies in Δ*cueA* mutant and Zn/Cd sensitive *E. coli* (Fig. 7). Very few studies so far have addressed the effect of P_1B1_-type ATPases on resistance to divalent metal ions. Intriguingly, an increase in Cu/Zn/Cd tolerance by overexpression of *cueA* in *csoR* deficient mutant was observed in this study (Fig. 6). CueA features two His-rich stretchs at its N-terminus, which is fundamentally distinct from the typical GXXCXXC motif in P_1B_-type ATPase (Fig. 3A). P_1B_-type ATPases with N-terminal His-rich stretch tend to transport divalent metal ion (Cu^2+^, Zn^2+^, Cd^2+^ and Pb^2+^), and the His-rich stretch is proposed to be a putative MBD^24^,^25^. In our complementation studies, deletion of the two His-rich MBD of CueA entirely abolished its function conferring Zn/Cd resistance in *E. coli* GG48 (Fig. 7B), showing the essential role of these amino acids in Zn/Cd resistance to the transporter. There were no previous reports describing the role of His-rich N-MBD of P_1B2_-type ATPase. However, the His-rich stretch in the C-terminus of plant Zn^2+^/Cd^2+^ transporting ATPases (TcHMA4 and AtHMA4) has been demonstrated experimentally and was shown to be essential for Zn^2+^/Cd^2+^ binding or in the regulation of the enzyme^39^. Lack of Zn/Cd sensitive phenotype in Δ*cueA* mutant may due to functional redundancy of CueA for the metals, in that multiple Zn^2+^/Cd^2+^ transporters systems are present in the genome of *B. liaoningense* CCNWSX0360 (LUKO00000000.1). This interpretation was supported by an unsuccessful screening for Zn sensitive mutant from the Tn*5*-induced mutant libraries.

Metal resistance might facilitate the survival of rhizobia in both free-living and symbiotic states in heavy metal-rich conditions. In our study, the *cueA*-deficient mutant showed the same nodulation capacity as the wild-type strain in the absence of Cu, suggesting that CueA is not essential for nodulation in normal conditions. However, in Cu rich conditions, the nodulation occupancy of the mutant decreased significantly (Fig. 8A). This is similar to the previous report that the nodulation of a Ni^2+^-sensitive mutant of *B. japonicum* was affected by the presence of nickel in soil^40^. Similarly, deletion of *dmeRF*, a Ni^2+^/Co^2+^ transport system, resulted in symbiotic performance defects of *Rhizobium leguminosarum* bv. *viciae* in high-cobalt conditions^35^. In addition, the decreased nodulation occupancy of the *cueA*-deficient mutant was Cu concentration-dependent, as it was less efficient in the presence of 500 mg/kg Cu^2+^ than 200 mg/kg Cu^2+^ (Fig. 8A). On the basis of these results, it is established that metal resistance determinants gives strains a competitive advantage in the establishment of symbiotic association with their host plants when faced with metal stress. The establishment of a symbiotic system is an intricate process, and how the metal(s) affect(s) the symbiosis remains unknown. With regard to the observed impairment in nodule occupancy efficiency in our Cu sensitive mutant, it is at least clear that Cu resistance is important for the symbiosis between *B. liaoningense* and *V. unguiculata* in Cu polluted soil. Further studies are required to elucidate the Zn/Cd resistance mechanism of CueA and the actual role of the Cu tolerance system in the symbiotic processes of these endosymbiotic bacteria under Cu stress.

## Methods

### Bacterial strains, media and growth conditions

Bacterial strains used in this study are listed in Table S1. Screening and identification of Cu resistant rhizobia were carried out as described in Supplementary Methods. *E. coli* strains including GG48 (∆*zitB*::Cm *zntA*::Km)^41^ were routinely cultured in LB medium at 37 °C. *B. liaoningense* strains were cultured in Tryptone-Yeast^42^ or YMA (Yeast-Mannitol Agar) medium at 28 °C^43^. Antibiotics were supplemented as required: streptomycin (Sm), 50 μg/ml; neomycin^24^, 100 μg/ml; kanamycin (Km), 50 μg/ml; ampicillin (Amp), 100 μg/ml; gentamicin (Gm), 25 μg/ml.

### Screening Cu sensitive Tn*5* mutant and determining the insertion sites

Transposon Tn*5* mutagenesis of Bln0360 was carried out using suicide plasmid pRL1063a according to the protocol for mutagenesis of *Rhizobium tropici* CIAT899^44^,^45^. Appropriate dilutions of the mating mixture were plated on YMA plates containing Sm and Amp. Individual colonies from YMA medium were picked and streaked onto TY plates supplemented with 0 and 0.8 mM CuSO_4_. Mutant clones that grew weakly or not at all on 0.8 mM Cu^2+^ plates but that grew well on control plates were selected for determination of Tn*5* insertion sites. To map the sequences contiguous with the inserted transposon, procedures including genomic DNA digestion, self-circularization, transformation, and sequencing were performed following published protocols^15^. Homology searches of nucleotide and deduced amino sequences were performed with the BLAST program (http://www.ncbi.nlm.nih.gov/blast).

### Deletion mutation and complementation of *csoR* and *cueA*

Plasmids used in the study were constructed as outlined in Supplementary Methods. Plasmid constructs carrying genes to be deleted (pK18mobsacB-Δ*cueA* and pK18mobsacB-Δ*csoR*) were introduced into Bln0360 by conjugal transfer using the *E. coli* S17-1 mobilizing strain according to the method of Simon *et al*.^46^. Gene deletion was achieved by sequential double crossover recombinants selection using YMA plate containing Neo/Amp and 10% sucrose as described previously^47^. To complement the Δ*csoR* and Δ*cueA* mutants, plasmids pBBR5-*csoR* and pBBR5-*cueA* respectively were electroporated into the Δ*csoR* and Δ*cueA* mutants. To analyze the role of the two N-terminal His-rich stretchs of CueA, plasmid constructs pJQ-1, pJQ-2 and pJQ-12 were transformed into Cu sensitive Δ*cueA* mutant and Zn sensitive *E. coli* GG48, respectively.

### Construction of chromosomal fusion reporter strains and β-galactosidase assays

The *lacZ* fusion reporter vectors (pK18mobSacB-P_*cueA*_::*lacZ* and pK18mobSacB-P_*csoR*_::*lacZ*; Table S1) were introduced into *E. coli* S17-1λpir and mated with Bln0360 and Δ*csoR* mutant strain, respectively^46^. Transconjugants were selected by plating on YMA plates supplemented with Neo and Amp and confirmed by PCR and sequencing. The *lacZ* fusion reporter strains were grown to mid-log phase in TY broth and then indicated inducers were added. After 2 h incubation, β-Galactosidase activity was measured using o-nitrophenyl-β-D-galactopyranoside as the substrate and was expressed in Miller units^48^.

### Quantitative real-time PCR and rapid amplification of cDNA ends

Total RNA was extracted from exponentially growing cell cultures using the hot phenol method^49^. First-strand cDNA was reverse transcribed using the PrimeScript^TM^ RT reagent kit with gDNA Eraser (Takara, Dalian, China). qRT-PCR was performed in a CFX96 Real-Time PCR Detection System (Bio-Rad, Hercules, CA USA) with a SYBR^®^ Premix Ex *Taq*™ II (Tli RNaseH Plus) kit (Takara). For all primer sets (Table S2), the following cycling parameters were used: 95 °C for 30 s, followed by 40 cycles of 94 °C for 15 s and 50 °C for 30 s. The relative abundance of 16S rRNA was used as the internal standard. To determine the initiating nucleotide for transcripts of *csoR* and *cueA*, the 5′ end of *csoR* and *cueA* mRNA were analyzed with the SMART RACE cDNA amplification kit (Clontech, Mountain View, CA, USA) according to the manufacturer’s protocols.

### Assays for tolerance to heavy metals

Log phase cultures of *B. liaoningense* or *E. coli* strains were washed twice and then 10-fold serially diluted in PBS buffer. An aliquot (3 μl) of dilution was spotted onto corresponding agar plates (TY or LB) containing varying concentrations of metal ions including Cu^2+^, Zn^2+^, Cd^2+^, Ni^2+^, or Ag^+^. Growth was monitored after 7 d at 28 °C for *B. liaoningense* and 36 h at 37 °C for *E. coli*. Dose-response growth curves describing the action of metal ions on bacterial cells were also performed. Log phase cultures were used to inoculate into parallel cultures containing increasing metal concentrations. The initial optical density of the cell suspension at 600 nm (OD_600_) was adjusted to 0.02. Cells were cultivated 7 d at 28 °C with shaking at 140 rpm for Bln0360 and 24 h at 37 °C with shaking at 200 rpm for *E. coli*, and the optical density was determined at 600 nm. Each experiment was repeated three times.

### Competition assay for nodulation

Nodule occupancy of the Δ*csoR* and Δ*cueA* strains in co-inoculations with Bln0360 were carried out as described by Patankar^50^. Briefly, surface-sterilized *V. unguiculata* seedlings were transplanted into pouches containing a sterilized mixture of vermiculite-perlite (2:1, v/v) supplemented with 0, 200 or 500 mg/kg CuSO_4_. After 2 days, individual plants were co-inoculated with 1 ml (10^6^ CFU) of inoculant combination containing the parental Bln0360 and either the Δ*cueA* or Δ*csoR* mutant in approximate 9:1, 1:1 and 1:9 ratios, with nine plants for each treatment. Seedlings without inoculation were used as negative controls. Plants were incubated in a controlled growth chamber (humidity: 70%; day condition: 22 °C, 16 h; night condition: 16 °C, 8 h). Fahraeus nitrogen-free nutrient solution was used to replenish the pouches, if required^51^. After 25 days, nodule samples from each treatment were surface sterilized and crushed in an appropriate volume of sterilized water as described by Shima^52^. Bacteria released from the crushed nodules were spotted onto YMA plates and the genetic backgrounds of the resultant colonies were confirmed by colony PCR with primer pair cueA-qc1/cueA-qc4. Significant difference analysis between the recovery ratio and initial inoculation ratio of the mutants was used to evaluate the competitive nodule occupancies^53^.

### Statistical analysis

Statistical analyzes were carried out using SPSS 19.0 software (SPSS Inc., Chicago, IL, USA). Paired two-tailed Student’s *t*-test was performed to determine significant differences among the treatments in transcription and β-galactosidase activity analysis. Statistical significance in competitive nodulation experiments was assessed by chi-square test at the significance level of *P* < 0.05.

## Additional Information

**How to cite this article**: Liang, J. *et al*. Functional characterization of a *csoR-cueA* divergon in *Bradyrhizobium liaoningense* CCNWSX0360, involved in copper, zinc and cadmium cotolerance. *Sci. Rep*. **6**, 35155; doi: 10.1038/srep35155 (2016).

## Supplementary Material

Supplementary Information

## Figures and Tables

**Table 1:** The location of transposon insertions in Bln0360 and the levels of metal tolerance in insertion mutants.

Strains/mutants	Gene^c^	Protein/putative function^a^^,^^c^	Maximum tolerable metal concentrations (MTC; mM)^b^						
			Cu^2+^	Zn^2+^	Cd^2+^	Pb^2+^	Ni^2+^	Co^2+^	Ag^+^
Bln0360	NA	NA	2.0	3.2	0.15	2.8	0.6	1.4	0.1
Bln-d	*cueA*	Heavy metal transporting P-type ATPase	0.8	3.2	0.15	2.8	0.6	1.4	0.1
Bln-163	*cueA*	Heavy metal transporting P-type ATPase	0.8	3.2	0.15	2.8	0.6	1.4	0.1
Bln-32	*tolC*	Type I secretion outer membrane protein	1.0	1.2	0.1	2.4	0.4	0.6	0.1
Bln-c	*copA*	Multicopper oxidase	0.6	3.2	0.15	2.8	0.6	1.4	0.1
Bln-29	*ctpA*	Carboxy-terminal protease, membrane integrity associated	0.6	2.8	0.1	1.6	0.6	1.4	0.1
Bln-54	*lptE*	Lipopolysaccharide-assembly lipoprotein	0.6	1.2	0.05	1.6	0.4	1.0	0.1

^a^Interrupted gene encoded proteins/functions are based on the annotated genome sequence of *B. diazoefficiens* USDA 110^17^.

^b^The MTCs were determined on TY plate containing elevated concentrations of metal ions (Cu^2+^/Co^2+^, 0–2.4 mM at 0.2 mM intervals; Zn^2+^, 0–4.0 mM at 0.4 mM intervals; Ag^+^, 0.025 0.05, 0.075, 0.1 and 0.125 mM; Ni^2+^, 0.2, 0.4, 0.6, and 0.8 mM).

^c^Not available.
