# Supplementary material for: Functional characterization of a csoR-cueA divergon in Bradyrhizobium liaoningense CCNWSX0360, involved in copper, zinc and cadmium cotolerance
Source: Sci Rep. 2016 Oct 11;6:35155. doi: 10.1038/srep35155 (PMC5057107; doi:10.1038/srep35155)
Supplement: Supplementary Information [file srep35155-s1.pdf]

Supplementary Information

Functional characterization of a *csoR-cueA* divergon in *Bradyrhizobium liaoningense* CCNWSX0360, involved in copper, zinc and cadmium cotolerance

Jianqiang Liang, Mingzhe Zhang, Mingmei Lu, Zhefei Li, Xihui Shen,

Minxia Chou*, Gehong Wei*

Supplementary Tables

Supplementary Table S1. Description of plasmids and bacterial strains used in this study

Plasmid or strain	Description	Source or reference
Bradyrhizobium liaoningense		
Bln0360	*Bradyrhizobium liaoningense* strain CCNWSX0360, Wild-type, Nod^+ on *Vigna unguiculata*, Cu^r, Sm^s, Neo^s, Amp^r	This study
Tn*5*-163	*cueA::*Tn*5* mutant of Bln0360; Cu^s, Sm^r	This study
Tn*5*-d	*cueA*::Tn*5* mutant of Bln0360; Cu^s, Sm^r	This study
Tn*5*-32	*tolC*::Tn*5* mutant of Bln0360; Cu^s, Sm^r	This study
Tn*5*-c	*copA*::Tn*5* mutant of Bln0360; Cu^s, Sm^r	This study
Tn*5*-29	*ctpA*::Tn*5* mutant of Bln0360; Cu^s, Sm^r	This study
Tn*5*-54	*lptE*::Tn*5* mutant of Bln0360; Cu^s, Sm^r	This study
Δ*csoR*	*csoR* deleted mutant of Bln0360	This study
Δ*cueA*	*cueA* deleted mutant of Bln0360	This study
Δ*cueA*(*cueA*)	Δ*cueA* containing pBBRMCS-5-*cueA*, Gm^r	This study
Δ*csoR*(*csoR*)	Δ*csoR* containing pBBRMCS-5-*csoR*, Gm^r	This study
P_{csoR}::*lacZ*	Chromosomally encoded P_{csoR}–*lacZ* translational fusion	This study
P_{cueA}::*lacZ*	Chromosomally encoded P_{cueA}–*lacZ* translational fusion	This study
Escherichia coli		
DH5α	F^- Φ80d *Lac*ZΔM15 *recA1 endA1 gyrA96* thi-1 *hsdR17* (rK^-, mK^+) *supE44 relA1 deoR* Δ(*lacZYA–argF*)U169	Stratagene
S17-1*λ pir*	*λ*-pir lysogen of S17-1, *thi pro hsdR* $hsdM^+$ *recA* RP42-Tc::Mu-Km::Tn7	[1]
GG48	Zn/Cd sensitive *E. coli*; Δ*zntA*::Km; Δ*zitB*::Cm	[2]
**Plasmids**		
pMD^{TM}18-T easy	Cloning and sequencing vector, Amp^r	Takara
pRK2013	Helper plasmid, Tra^+, Km^r	[3]
pRL1063a	Mobilizable suicide plasmid for Tn*5* mutagenesis carrying Tn*5*-*luxAB*, Km^r, Sm^r	[4]
pK18mobsacB	Allelic exchange suicide vector mobilized by *E. coli* S17-1 *λpir*, *sacB*, *lacZα*, *mcs*, Km^r (Neo^r)/mobilizable	[5]
pBBRMCS-5	Mobilizable broad-host-range vector; Gm^r	[6]

Supplementary Table S2. Primers used in this study

Primers	5′-3′ sequence	Function
P1	AGAGTTTGATCCTGGCTCAGAACGAACGCT	Amplification of
P6	TACGGCTACCTTGTTACGACTTCACCCC	16S rRNA
Tn-F	TACTAGATTCAATGCTATGAATGAG	For sequencing
Tn-R	AGGAGGTCACAGGAATATCAGAT	self-ligated pRL1063a
cueA-qc1	CGGAATTCCGCGCGATTCCTCGCTCTACATG (*Xba*I)	To generate
cueA-qc2	aaggtgggatagaggacgccTGACGATGCTCGCTGCTGTT	pK18mobsacB-Δ*cueA*
cueA-qc3	GGCGTCCTCTATCCCACCTT	
cueA-qc4	CCCAAGCTTGGCGATCCAGGACAGGACTTCGA (*Pst*I)	
csoR-qc1	AACAGATGCCTTGATGTCCTTGC	To generate
csoR-qc2	CGGAATTCCGTCGCGATCAGCGTGAACATG (*Eco*RI)	pK18mobsacB-Δ*csoR*
csoR-qc3	GCTCTAGAGCTGCGTTATCTGTTCCCGATGC (*Xba*I)	
csoR-qc4	gcaaggacatcaaggcatctgttCGGAGCTGATGGCGGTGAT	
csoR-HB1	GGGGTACCCATGCGCAAGGACATCAAGGCATC (*Kpn*I)	To generate
csoR-HB2	GCTCTAGAGC AATGACGGTGGAGAGAGCGTG (*Xba*I)	pBBRMCS-5-*csoR*
cueA-HB1	CCGCTCGAGCGGTATGAACAGCAGCGAGCATCGTC (*Xho*I)	To generate pBBRMCS-5-*cueA*
cueA-HB2	GCTCTAGAGCTCACCGGGCTGTTGTAGCCTTTCC (*Xba*I)	
P_{csoR}-F	GCTCTAGAGC AGCTTTCGTGGAACAGCCGC (*Xba*I)	To generate
P_{csoR}-R	AACTGCAGAACCAATGCATTGCGATGTCGATGCAGTAGCGG (*Pst*I)	pK18mobsacB-P_{csoR}
P_{cueA}-F	GCTCTAGAGC CGATGTCGATGCAGTAGCGG (*Xba*I)	To generate
P_{cueA}-R	AACTGCAGAACCAATGCATTGGAGCTTTCGTGGAACAGCCGC (*Pst*I)	pK18mobsacB-P_{cueA}
Race-cueA	GGGTGCATCGGACAGGTGTAG	5’ Race for *cueA*
Race-csoR	ATCACCGCCATCAGCTCCG	5’ Race for *csoR*
qRTcsoR-F	ATCACCGCCATCAGCTCCG	qRT-PCR for *csoR*
qRTcsoR-R	ACCGCTACTGCATCGACATCGT	
qRTcueA-F	GAACAGCAGCGAGCATCGTCAC	qRT-PCR for *cueA*
qRTcueA-R	CGCAAACCGGGTCCTTCACC	
qRT16S rRNA-F	CCACTAACGGCTGGCATTCATCG	qRT-PCR for 16S rRNA
qRT16S rRNA-R	TTGCTCAATTCGTCGCTCCATT	
cueA-His-1F	CCGGAATTCCGGGCCGCCGCAGCACGAGGATGC (*Eco*RI)	For the first His-rich
cueA-His-1R	CCGGAATTCCGGGATGCCGCGACCAGGGTGAAG (*Eco*RI)	region deletion
cueA-His-2F	CCGGAATTCCGGCTTCGAGGTCGCGGGATCGAC (*Eco*RI)	For the second His-rich
cueA-His-2R	CCGGAATTCCGGTGCTCGGCCGGCTGCCGCAC (*Eco*RI)	region deletion

The primer sequences are shown from 5′ to 3′. Restriction sites incorporated into the primers are underlined and the name of the restriction enzymes indicated in parentheses. Complementary sequence designed for SOE-PCR (splicing by overlapping extension polymerase chain reaction) is shown in lowercase letters.

Supplementary Figures

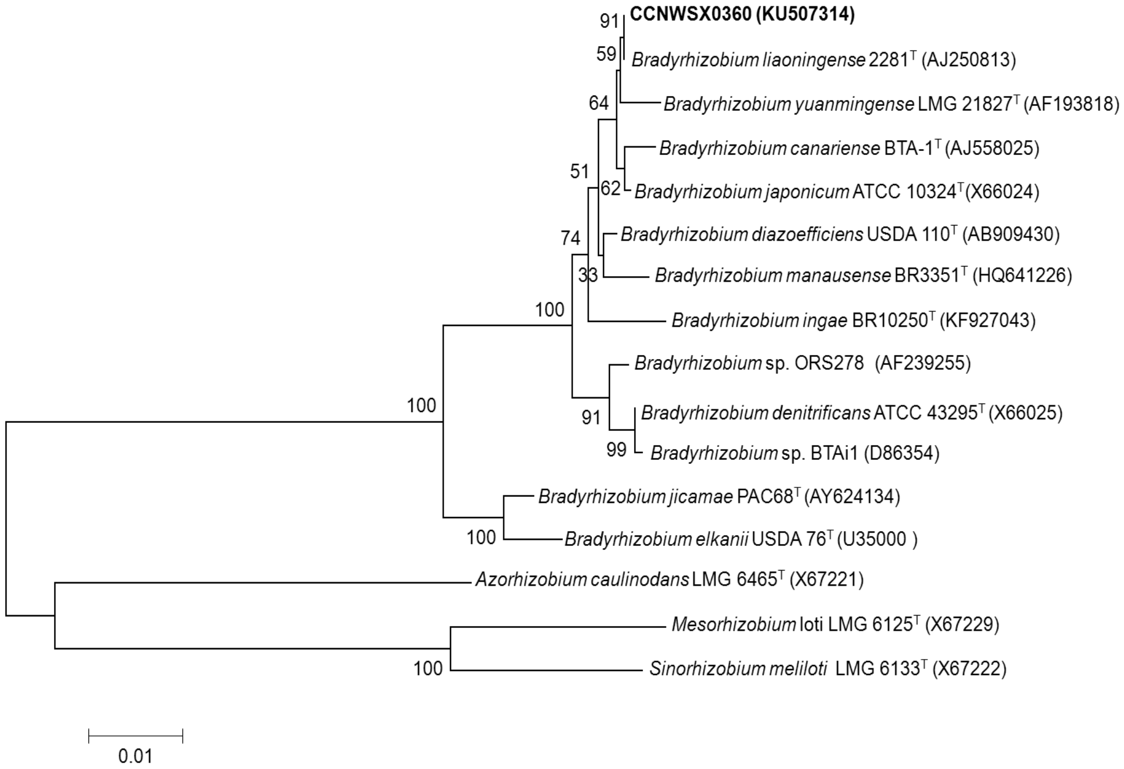

Supplementary Fig. S1. A phylogenetic tree showing the position of *B. liaoningense* strain CCNWSX0360 (Bln0360) based on 16S rRNA sequences. GenBank accession number of each 16S rRNA gene is indicated in parentheses. Bootstrap values are obtained from 1,000 replications. Bar represents 0.01 substitutions per nucleotide position.

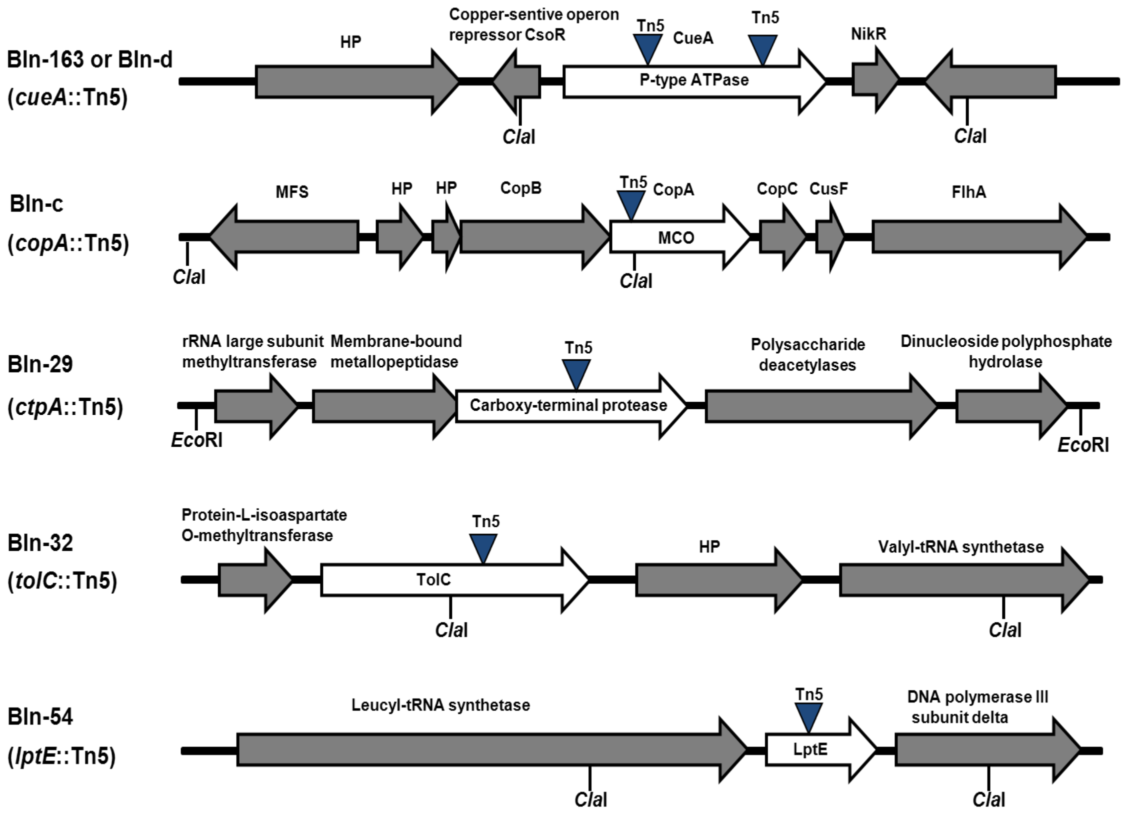

Supplementary Fig. S2. Schematics of genes interrupted by Tn*5* transposon. Vertical arrows indicate the location of Tn*5* transposons. Genes flanking these loci and their deduced protein are also shown. HP means hypothetical protein. The restriction enzymes used for digesting the genomic DNA of each Tn*5* inserted mutant are listed below the gene set.

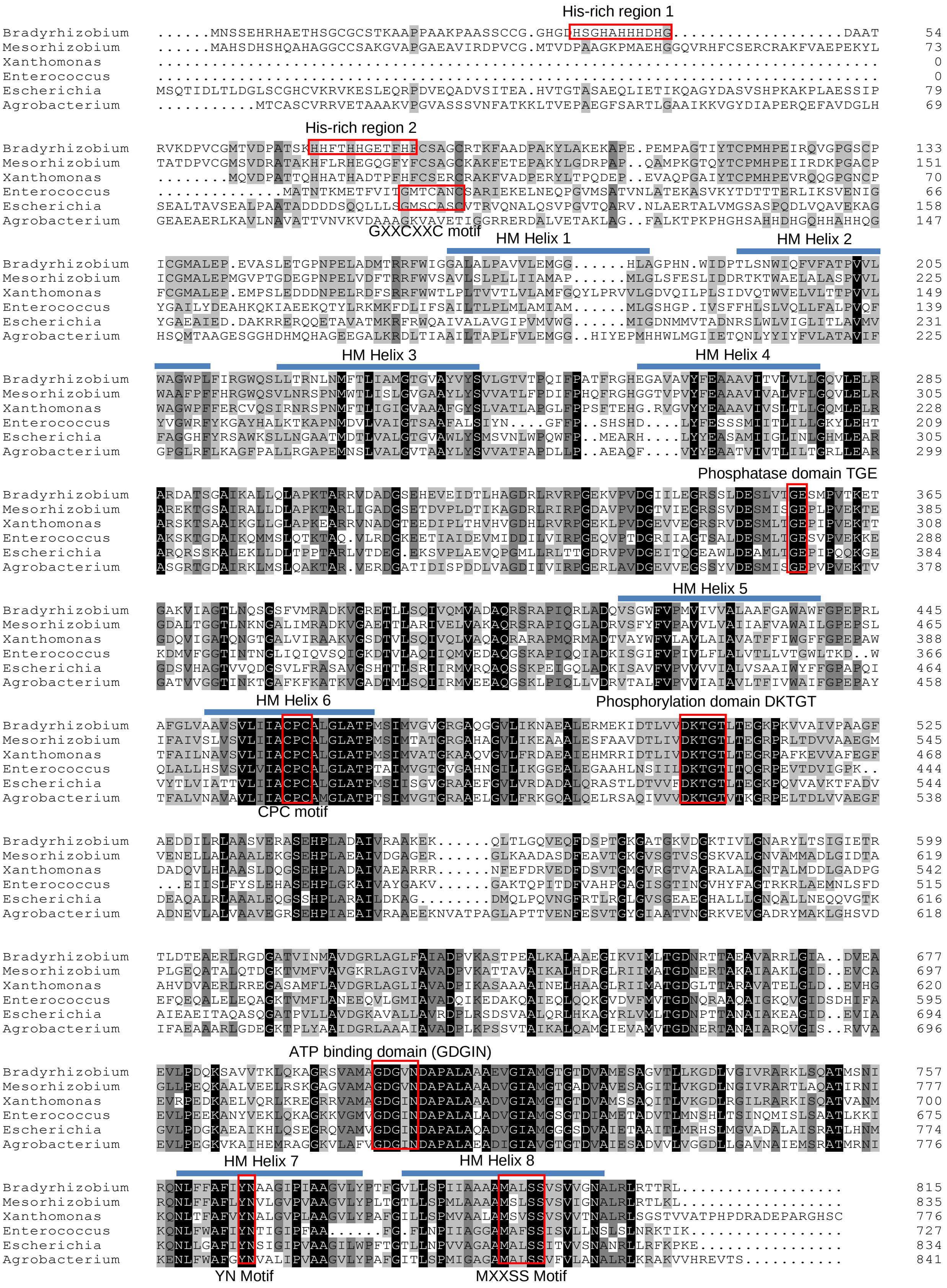

His-rich region 1
His-rich region 2
GXXCXXC motif
HM Helix 1
HM Helix 2
HM Helix 3
HM Helix 4
Phosphatase domain TGE
HM Helix 5
HM Helix 6
Phosphorylation domain DKTGT
CPC motif
ATP binding domain (GDGIN)
HM Helix 7
HM Helix 8
YN Motif
MXXSS Motif
Bradyrhizobium
Mesorhizobium
Xanthomonas
Enterococcus
Escherichia
Agrobacterium

Supplementary Fig. S3. Amino acid sequence alignment of CueA homologues protein from *Mesorhizobium amorphae* (EHH02252)[7], *Xanthomonas vesicatoria* (WP_005995730), *Escherichia coli* (Q59385)[8], *Enterococcus hirae* (P32113)[9], and *Agrobacterium tumefaeiens* (Atu0937)[10]. Sequence alignments were carried out using CLUSTAL W2 program, included in the DNAMAN software package (Version 5.2.2). The positions of the last residue in the aligned fragments are listed in the right column. The conserved domains, metal binding motif, and the signature sequences are boxed and indicated above the boxes.

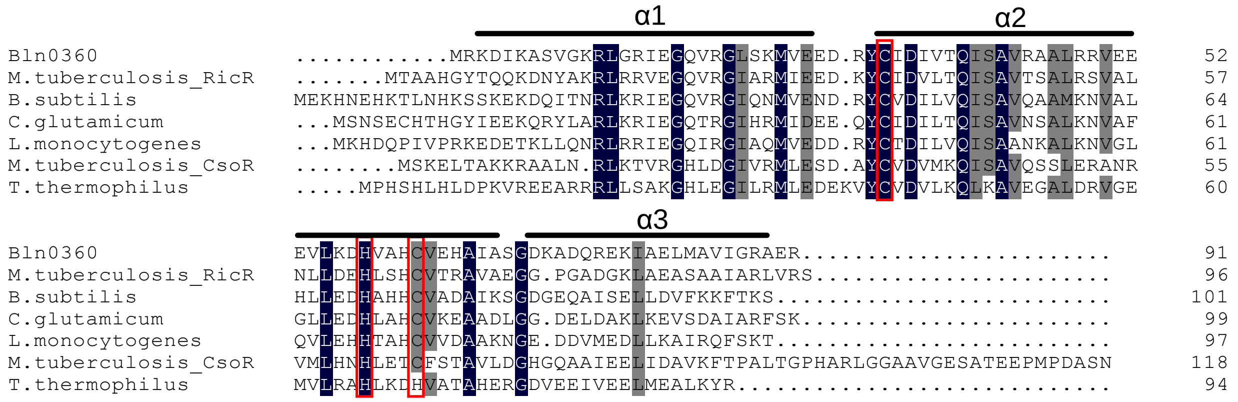

Supplementary Fig. S4. Amino acid sequence alignment for homologues proteins of *B. liaoningense* CsoR. The sequences used for alignments were retrieved from *Thermus thermophilus* (BAF68263)[11], *Mycobacterium tuberculosis* CsoR (P9WP49)[12], *Corynebacterium glutamicum* (AIK84123)[13], *M. tuberculosis* RicR (NP_214704)[14], *Listeria monocytogenes* (CAC99932)[15], and *Bacillus subtilis* (O32222)[16]. The conserved Cu^{+}-coordinating residues Cys, His, and Cys are marked with red box. The sequences were aligned using CLUSTAL W2 program. The right numbers represent the positions of the residue correspond to the initial methionine of CsoR. Three predicted TM α-helices are labeled with thick lines above the aligned sequences.

Supplementary Methods:

Screening and identification of Cu resistant rhizobia. Bacteria were isolated using Vincent's method from the nodules of 13 leguminous plant species including *Medicago lupulina*, *Trifolium repens*, *Robinia pseudoacacia*, and *Vigna unguiculata*. The plants grew in Cu/Zn mine tailing and surrounding areas in Ningqiang County, Shannxi Province, northwestern China. Cu resistant isolates were screened using TY agar medium as described by Hao et al.[17]. The maximum tolerable concentration (MTC) was determined using TY agar medium containing a series of concentrations of different metal ions and defined as the highest concentration of a metal at which the growth of isolates could be observed[18]. For identification, the nearly full-length 16S rRNA gene was amplified from bacterial genomic DNA using primer pair P1/P6[17]. Multiple alignments of 16S rRNA gene sequences were carried out using the CLUSTAL W program[19]. A phylogenetic tree was constructed by the neighbor-joining method (Kimura two-parameter model) using MEGA 5.0 (www.megasoftware.net), with 1,000 replicates for bootstrap analysis.

Plasmid construction. Primers used in this study are listed in Table S2. To construct the Δ*cueA* in-frame deletion mutant of Cu resistant *B. liaoningense* CCNWSX0360 (Bln0360), 873-bp and 797-bp fragments flanking *cueA* were amplified using primer pairs cueA-qc1/cueA-qc2 and cueA-qc3/cueA-qc4, respectively. The upstream and downstream PCR fragments were ligated by overlap PCR[20]. The resulting amplicon was digested with *Xba*I and *Pst*I, and then inserted into similarly digested pK18mobsacB[5] to generate pK18mobsacB-*cueA*. The same method was used to construct the plasmid pK18mobsacB-Δ*csoR* with primer pairs csoR-qc1/csoR-qc2 and csoR-qc3/csoR-qc4.

To complement the *cueA* deletion mutant, primers cueA-HB1 and cueA-HB2 were used to amplify full-length *cueA* from the genomic DNA of strain Bln0360. The PCR product was digested with *Xba*I/*Xho*I and inserted into similarly digested pBBRMCS-5 to produce pBBR5-*cueA*[6]. Plasmid pBBR5-*csoR* was constructed in the same manner using primers csoR-HB1/csoR-HB2.

For construction of *lacZ* fusion reporter strains, a promoterless *lacZ* fragment amplified from genomic DNA of *E. coli* K-12 with primers lacZ-F/lacZ-R was cloned into pK18mobSacB, generating pK18mobSacB::*lacZ*. Then, a 232-bp fragment containing the *csoR*-*cueA* intergenic region was amplified using primers P_{csoR}-F and P_{csoR}-R. After digestion with *Xba*I/*Pst*I, the PCR product was cloned into pK18mobSacB::*lacZ*, producing plasmid pK18mobSacB-P_{csoR}::*lacZ*. To

create pK18mobSacB-P$_{cueA}$::*lacZ*, primers P$_{cueA}$-F and P$_{cueA}$-R were used and the PCR product was cloned into pK18mobSacB::*lacZ*.

To investigate the role of N-terminal His-rich stretch of CueA, a plasmid used for expression of CueA variants was constructed based on inverse PCR using plasmid pBBR5-*cueA* as the template. Briefly, for deletion of the first His-rich stretch, the PCR product amplified with primer pair cueA-his-1F/cueA-his-1R was digested with *Eco*RI followed by a self-circularization generating pJQ-1. Plasmid pJQ-2 used to delete the second His-rich stretch was constructed similarly using primers cueA-His-2F and cueA-His-2R. Plasmid pJQ-12 used to delete both His-rich stretchs was constructed using primers cueA-His-2F and cueA-His-2R only with plasmid pJQ-1 as the template. The fidelity and correctness of reading frame of all constructs were confirmed by DNA sequencing.

References

1 Simon, R., Priefer, U. & Pühler, A. A broad host range mobilization system for invivo genetic-engineering-transposon mutagenesis in gram-negative bacteria. *Bio-technology* **1**, 784-791 (1983).

2 Grass, G. *et al.* ZitB (YbgR), a Member of the Cation Diffusion Facilitator Family, Is an Additional Zinc Transporter inEscherichia coli. *J. Bacteriol.* **183**, 4664-4667 (2001).

3 Figurski, D. H. & Helinski, D. R. Replication of an origin-containing derivative of plasmid RK2 dependent on a plasmid function provided in trans. *Proc. Natl. Acad. Sci. USA* **76**, 1648-1652 (1979).

4 Wolk, C. P., Cai, Y. & Panoff, J.-M. Use of a transposon with luciferase as a reporter to identify environmentally responsive genes in a cyanobacterium. *Proc. Natl. Acad. Sci. USA* **88**, 5355-5359 (1991).

5 Schäfer, A. *et al.* Small mobilizable multi-purpose cloning vectors derived from the *Escherichia coli* plasmids pK18 and pK19: selection of defined deletions in the chromosome of *Corynebacterium glutamicum*. *Gene* **145**, 69-73 (1994).

6 Kovach, M. E. *et al.* Four new derivatives of the broad-host-range cloning vector pBBR1MCS, carrying different antibiotic-resistance cassettes. *Gene* **166**, 175-176 (1995).

7 Hao, X. *et al.* Copper tolerance mechanisms of *Mesorhizobium amorphae* and its role in aiding phytostabilization by *Robinia pseudoacacia* in copper contaminated soil. *Environ. Sci. Technol.* **49**, 2328-2340 (2015).

8 Rensing, C., Fan, B., Sharma, R., Mitra, B. & Rosen, B. P. CopA: an *Escherichia coli* Cu(I)-translocating P-type ATPase. *Proc. Natl. Acad. Sci. USA* **97**, 652-656 (2000).

9 Odermatt, A., Krapf, R. & Solioz, M. Induction of the putative copper ATPases, CopA and CopB, of *Enterococcus hirae* by Ag^+ and Cu^{2+}, and Ag^+ extrusion by CopB. *Biochem. Biophys. Res. Commun.* **202**, 44-48 (1994).

10 Nawapan, S. *et al.* Functional and expression analyses of the *cop* operon, required for copper resistance in *Agrobacterium tumefaciens*. *J. Bacteriol.* **191**, 5159-5168 (2009).

11 Sakamoto, K., Agari, Y., Agari, K., Kuramitsu, S. & Shinkai, A. Structural and functional characterization of the transcriptional repressor CsoR from *Thermus thermophilus* HB8. *Microbiology* **156**, 1993-2005 (2010).

12 Liu, T. *et al.* CsoR is a novel *Mycobacterium tuberculosis* copper-sensing transcriptional regulator. *Nat. Chem. Biol.* **3**, 60-68 (2007).

13 Teramoto, H., Yukawa, H. & Inui, M. Copper homeostasis-related genes in three separate transcriptional units regulated by CsoR in *Corynebacterium glutamicum*. *Appl. Microbiol. Biotechnol.* **99**, 3505-3517 (2015).

14 Shi, X. *et al.* The copper-responsive RicR regulon contributes to *Mycobacterium tuberculosis* virulence. *MBio* **5**, e00876-00813 (2014).

15 Corbett, D. *et al.* The combined actions of the copper-responsive repressor CsoR and copper-metallochaperone CopZ modulate CopA-mediated copper efflux in the intracellular pathogen *Listeria monocytogenes*. *Mol. Microbiol.* **81**, 457-472 (2011).

16 Smaldone, G. T. & Helmann, J. D. CsoR regulates the copper efflux operon *copZA* in *Bacillus subtilis*. *Microbiology* **153**, 4123-4128 (2007).

17 Hao, X. *et al.* Genome sequence and mutational analysis of plant-growth-promoting bacterium *Agrobacterium tumefaciens* CCNWGS0286 isolated from a zinc-lead mine tailing. *Appl. Environ. Microbiol.* **78**, 5384-5394 (2012).

18 Duffus, J. Glossary for chemists of terms used in toxicology (IUPAC Recommendations 1993). *Pure Appl. Chem.* **65**, 2003-2122 (1993).

19 Aiyar, A. The use of CLUSTAL W and CLUSTAL X for multiple sequence alignment. *Bioinformatics methods and protocols*, 221-241 (1999).

20 Heckman, K. L. & Pease, L. R. Gene splicing and mutagenesis by PCR-driven overlap extension. *Nat Protoc* **2**, 924-932 (2007).
